# Supplementary figures and images for: Lactate is an energy substrate for rodent cortical neurons and enhances their firing activity
Source: eLife. 2021 Nov 12;10:e71424. doi: 10.7554/eLife.71424 (PMC8651295; doi:10.7554/eLife.71424)

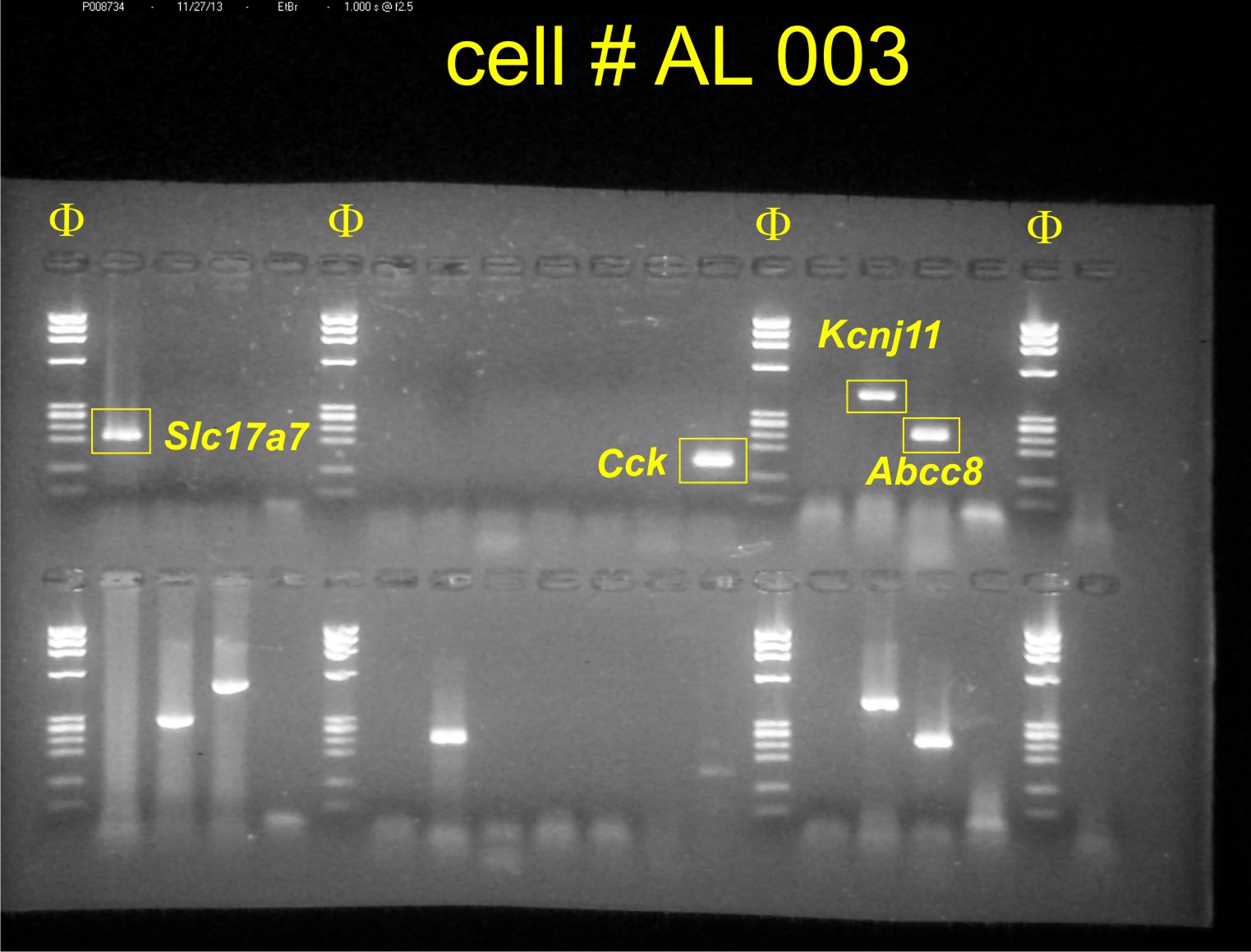

Supplement: Figure 1—source data 3. [file elife-71424-fig1-data3.zip › Figure1 - source data 3.jpg]

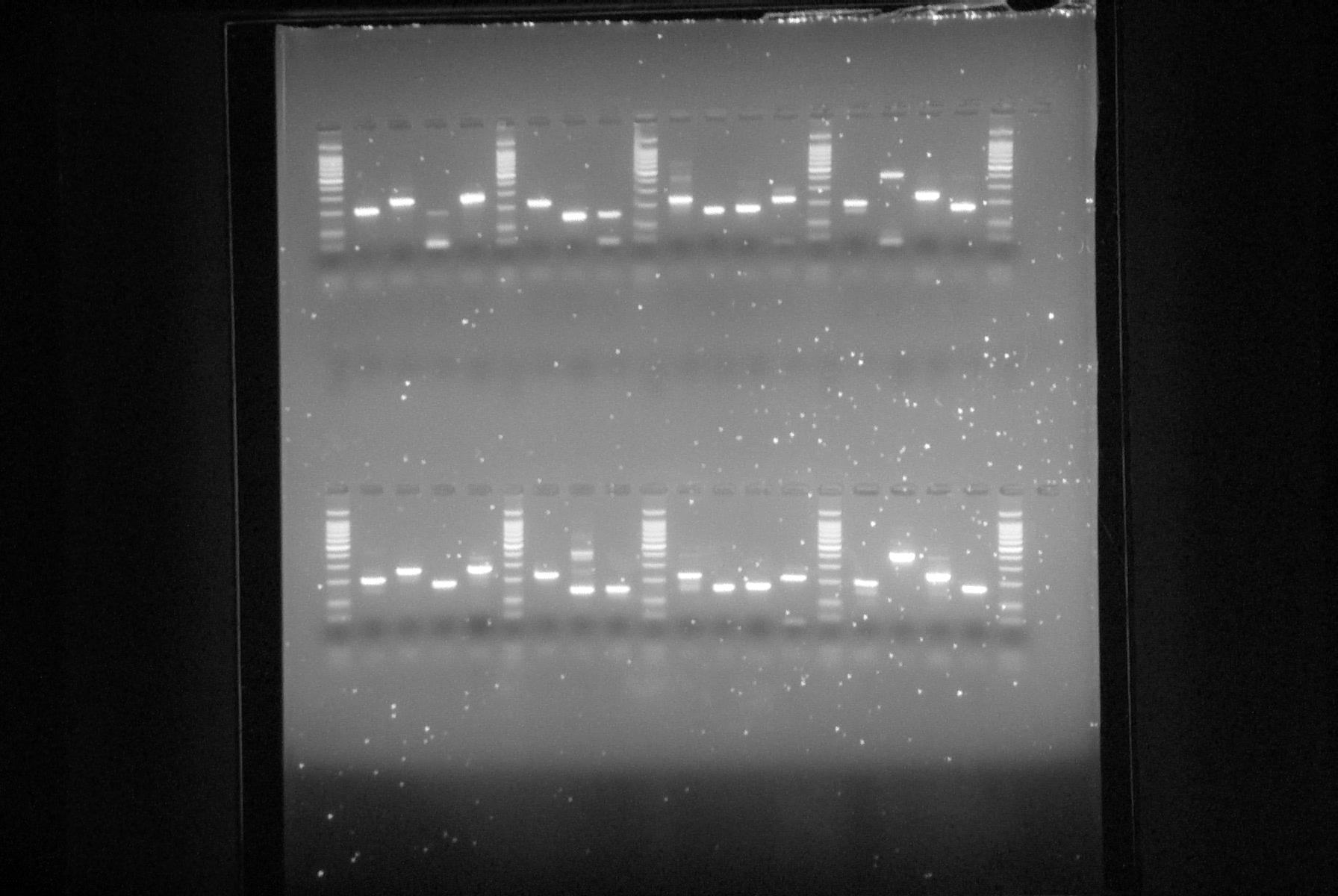

Supplement: Figure 1—figure supplement 1—source data 1. [file elife-71424-fig1-figsupp1-data1.zip › Figure1 -figure supplement 1-source data 1.jpg]

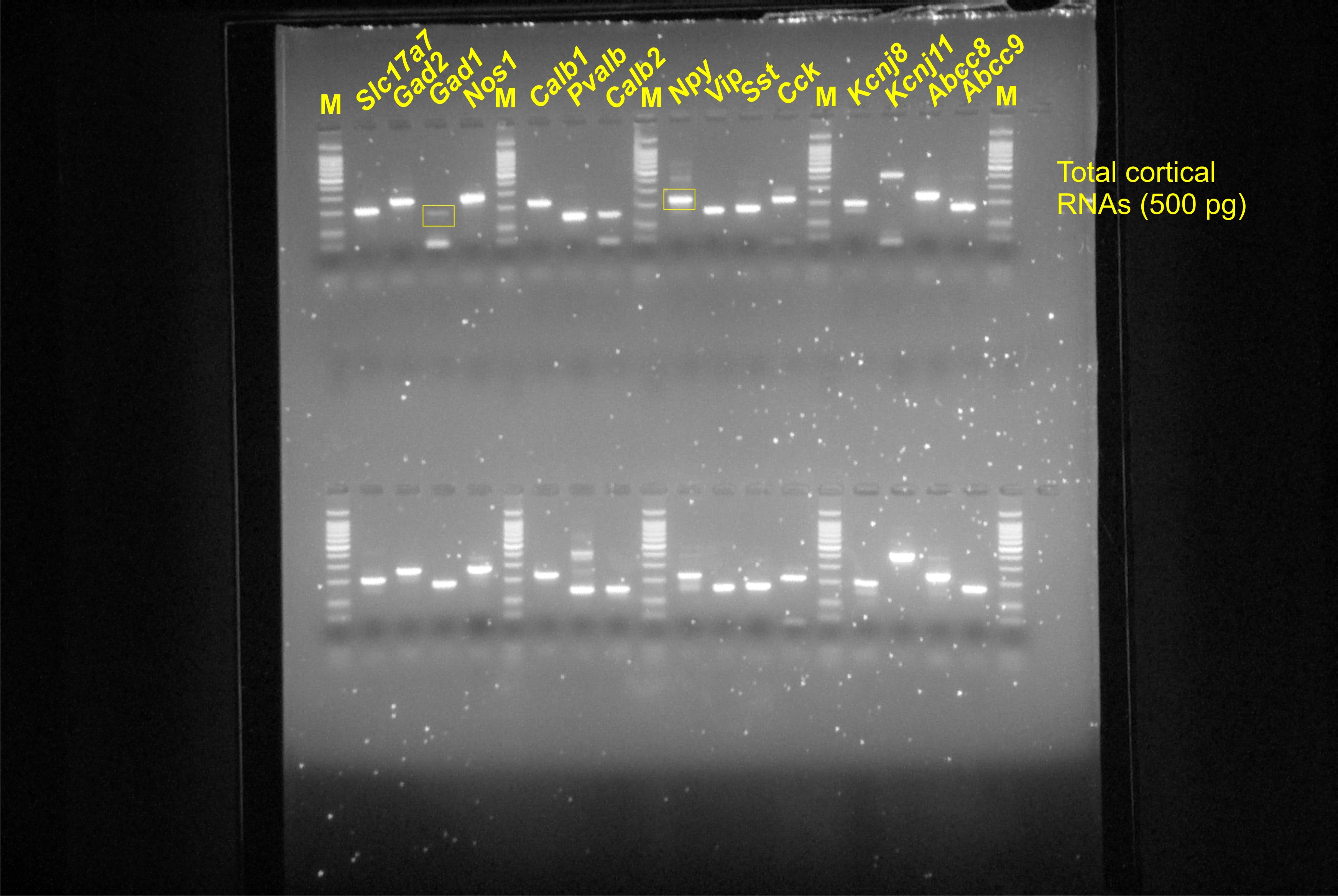

Supplement: Figure 1—figure supplement 1—source data 2. — Yellow rectangles denote bands of the expected size. [file elife-71424-fig1-figsupp1-data2.zip › Figure1 -figure supplement 1-source data 2.jpg]

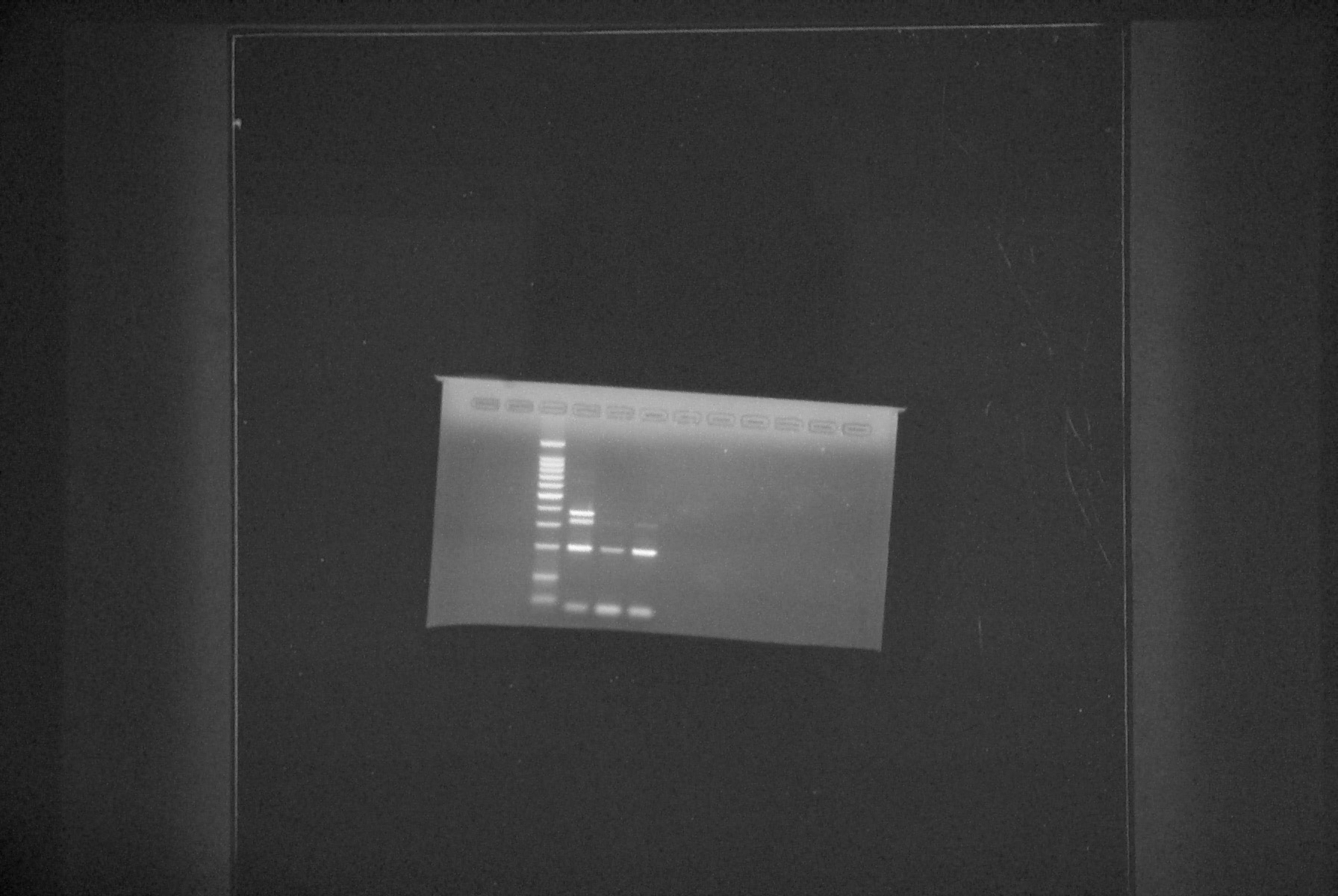

Supplement: Figure 1—figure supplement 1—source data 3. [file elife-71424-fig1-figsupp1-data3.zip › Figure1 -figure supplement 1-source data 3.jpg]

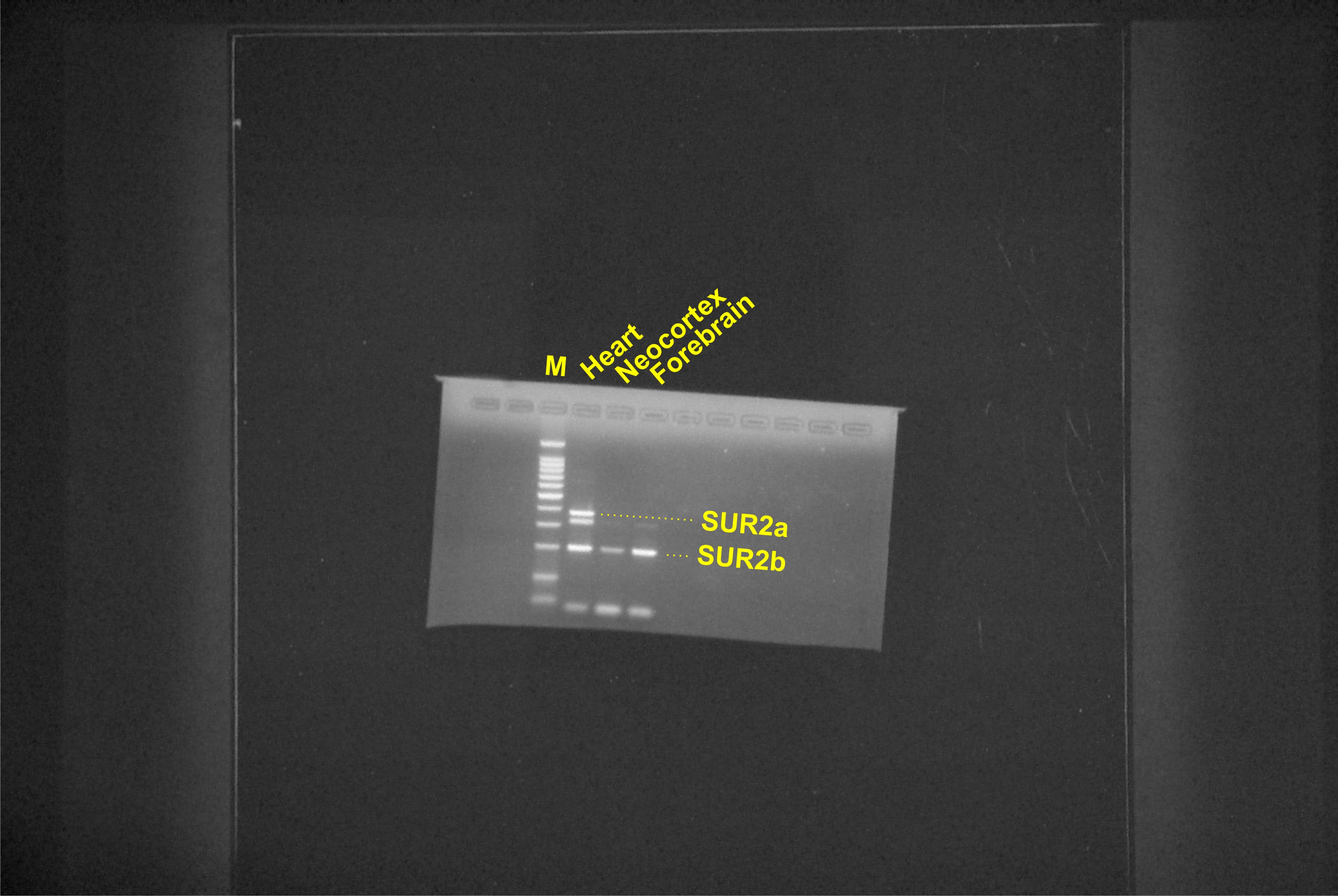

Supplement: Figure 1—figure supplement 1—source data 4. [file elife-71424-fig1-figsupp1-data4.zip › Figure1 -figure supplement 1-source data 4.jpg]
