# Supplementary material for: Lactate is an energy substrate for rodent cortical neurons and enhances their firing activity
Source: eLife. 2021 Nov 12;10:e71424. doi: 10.7554/eLife.71424 (PMC8651295; doi:10.7554/eLife.71424)
Supplement: Supplementary file 1. — n.s., not statistically significant. [file elife-71424-supp1.doc]

**Supplementary file 1. Somatic properties of different neuronal types**

|  | **RS** | **IB** | **Burst. *Vip*** | **Adapt. *Vip*** | **Adapt. *Sst*** | **Adapt. *Npy*** | **FS-*Pvalb*** |
| --- | --- | --- | --- | --- | --- | --- | --- |
|  | **n = 63** | **n = 10** | **n = 27** | **n = 59** | **n = 24** | **n = 56** | **n = 38** |
| **Layer** | 2.9 ± 0.1 | **3.9 ± 0.1** | 2.5 ± 0.1 | 2.6 ± 0.1 | 3.0 ± 0.1 | **2.1 ± 0.1** | 3.1 ± 0.1 |
|  | **Adapt. Npy** <<< RS, IB, Adapt. *Vip*, Adapt. *Sst*, FS-*Pvalb*  Burst. *Vip,* Adapt. *Vip* <<< **IB**; Adapt. *Sst* << **IB**; RS < **IB** | | | | | | |
| **Major axis (µm)** | 22.3 ± 1.2 | 16.7 ± 1.3 | 17.8 ± 0.9 | 20.5 ± 0.6 | 24.7 ± 1.6 | 21.2 ± 1.1 | 22.9 ± 1.1 |
|  | IB < Adapt. *Sst*, FS-*Pvalb* | | | | | | |
| **Minor axis (µm)** | 11.5 ± 0.5 | 9.2 ± 0.3 | 9.2 ± 0.3 | 9.0 ± 0.2 | 9.4 ± 0.3 | 9.6 ± 0.2 | 9.8 ± 0.3 |
|  | Adapt. *Vip* <<< RS | | | | | | |
| **Elongation** | 2.0 ± 0.1 | 1.8 ± 0.2 | 2.0 ± 0.1 | 2.3 ± 0.1 | 2.6 ± 0.2 | 2.2 ± 0.1 | 2.3 ± 0.1 |
|  | RS << Adapt. *Vip*, Adapt. *Sst*; RS < FS-*Pvalb*  IB < Adapt. *Vip*, Adapt. *Sst*, FS-*Pvalb* | | | | | | |
| **Area (µm^2^)** | 157.3 ± 8.3 | 115.4 ± 6.9 | 116.0 ± 6.4 | 127.0 ± 4.2 | 161.6 ± 10.9 | 145.0 ± 7.6 | 160.9 ± 7.0 |
|  | Burst. *Vip*, Adapt. *Vip* <<< FS-*Pvalb*; IB < FS-*Pvalb*  Burst. *Vip* << Adapt. *Sst*; IB, Adapt. *Vip* < Adapt. *Sst*  Burst. *Vip* << RS | | | | | | |
| **Perimeter (µm)** | 58.0 ± 2.9 | 44.2 ± 2.3 | 44.7 ± 1.8 | 48.8 ± 1.2 | 58.0 ± 3.3 | 52.1 ± 2.1 | 54.9 ± 2.5 |
|  | IB, Burst. *Vip*, Adapt. *Vip* < FS-*Pvalb* | | | | | | |
| **Roundness** | 1.7 ± 0.1 | 1.4 ± 0.1 | 1.4 ± 0.0 | 1.6 ± 0.1 | 1.7 ± 0.1 | 1.5 ± 0.0 | 1.6 ± 0.1 |
|  | n.s. | | | | | | |

n, number of cells, < significantly smaller with P ≤ 0.05; << significantly smaller with P ≤ 0.01; <<< significantly smaller with P ≤ 0.001. . n.s. not statistically significant.
