# Supplementary material for: Lactate is an energy substrate for rodent cortical neurons and enhances their firing activity
Source: eLife. 2021 Nov 12;10:e71424. doi: 10.7554/eLife.71424 (PMC8651295; doi:10.7554/eLife.71424)
Supplement: Supplementary file 2. — Detection rates are given in %; n, number of cells; > significantly larger with p ≤ 0.05; >> significantly larger with p ≤ 0.01; >>> significantly larger with p ≤ 0.001. n.s., not statistically significant. [file elife-71424-supp2.docx]

**Supplementary file 2. Detection rate of molecular markers in different neuronal types**

|  | **RS** | **IB** | **Burst. Vip** | **Adapt. Vip** | **Adapt. Sst** | **Adapt. Npy** | **FS-Pvalb** |
| --- | --- | --- | --- | --- | --- | --- | --- |
|  | **n = 63** | **n = 10** | **n = 27** | **n = 59** | **n = 24** | **n = 56** | **n = 38** |
| ***Slc17a7*** | **94** | **100** | **44** | **17** | **33** | **34** | **32** |
|  | **RS** >>>Burst. *Vip*, Adapt. *Vip*, Adapt. *Sst*, Adapt. *Npy*, FS-*Pvalb*  **IB** >>> Adapt. *Vip*; **IB** >> Adapt. *Sst*, Adapt. *Npy*, FS-*Pvalb*; **IB** > Burst. *Vip* | | | | | | |
| ***Gad*** | **10** | **10** | **100** | **100** | **100** | **96** | **100** |
|  | **Burst. *Vip*, Adapt. *Vip*, Adapt. *Sst*, Adapt. *Npy*, FS,*Pvalb*** >>> RS, IB | | | | | | |
| ***Nos1*** | **0** | **0** | **0** | **5** | **13** | **21** | **8** |
|  | **Adapt. *Npy*** >> RS | | | | | | |
| ***Calb1*** | **40** | **40** | **7** | **17** | **88** | **11** | **50** |
|  | **Adapt. *Sst*** >>> Burst. *Vip*, Adapt. *Vip*, Adapt. *Npy*; **Adapt. *Sst*** >> RS, **Adapt. *Sst*** > FS-*Pvalb*  FS-*Pvalb* >>> Adapt. *Npy*; FS-*Pvalb* >> Burst. *Vip*, Adapt. *Vip*  RS >> Adapt. *Npy*, RS > Burst. *Vip* | | | | | | |
| ***Pvalb*** | **33** | **20** | **11** | **20** | **38** | **21** | **97** |
|  | **FS-*Pvalb*** >>> RS, IB, Burst. *Vip*, Adapt. *Vip*, Adapt. *Npy*; Adapt. *Sst* | | | | | | |
| ***Calb2*** | **0** | **0** | **26** | **39** | **4** | **16** | **3** |
|  | **Adapt. *Vip*** >>> RS, FS-*Pvalb*, Adapt. *Vip* > Adapt. *Sst*  Burst. *Vip* >> RS, Adapt. *Npy* > RS | | | | | | |
| ***Npy*** | **0** | **10** | **7** | **12** | **67** | **55** | **24** |
|  | **Adapt. *Sst*, Adapt. *Npy*** >>> RS, Burst. *Vip*, Adapt. *Vip*; **Adapt. *Sst*, Adapt. *Npy*** > FS-*Pvalb*  FS-*Pvalb* >> RS | | | | | | |
| ***Vip*** | **0** | **0** | **89** | **83** | **4** | **11** | **3** |
|  | **Burst. *Vip*, Adapt. *Vip*** >>> RS, IB, Adapt. *Sst*, Adapt. *Npy*, FS-*Pvalb* | | | | | | |
| ***Sst*** | **10** | **0** | **11** | **8** | **92** | **9** | **5** |
|  | **Adapt. *Sst*** >>> RS, IB, Burst. *Vip*, Adapt. *Vip*, Adapt. *Npy*, FS-*Pvalb* | | | | | | |
| ***Cck*** | **33** | **10** | **22** | **34** | **4** | **18** | **8** |
|  | n.s. | | | | | | |

Detection rates are given in %; n, number of cells; > significantly larger with P ≤ 0.05; >> significantly larger with P ≤ 0.01; >>> significantly larger with P ≤ 0.001. n.s. not statistically significant.
