# Supplementary material for: Lactate is an energy substrate for rodent cortical neurons and enhances their firing activity
Source: eLife. 2021 Nov 12;10:e71424. doi: 10.7554/eLife.71424 (PMC8651295; doi:10.7554/eLife.71424)
Supplement: Supplementary file 3. [file elife-71424-supp3.docx]

**Supplementary file 3. Passive properties of different neuronal types**

|  | **RS** | **IB** | **Burst. *Vip*** | **Adapt. *Vip*** | **Adapt. *Sst*** | **Adapt. *Npy*** | **FS-*Pvalb*** |
| --- | --- | --- | --- | --- | --- | --- | --- |
|  | **n = 63** | **n = 10** | **n = 27** | **n = 59** | **n = 24** | **n = 56** | **n = 38** |
| **(1) Resting potential (mV)** | **-81.5 ± 0.9** | -79.2 ± 1.1 | -75.6 ± 1.2 | -73.9 ± 0.8 | **-71.6 ± 1.1** | -76.6 ± 0.7 | -77.4 ± 0.9 |
|  | RS <<< Adapt. *Vip*, Adapt. *Sst*; RS << Adapt. *Npy*; RS < Burst. *Vip*, FS-*Pvalb*  IB, Adapt. *Npy* << Adapt. *Sst*; FS-*Pvalb* <<< Adapt. *Sst* | | | | | | |
| **(2) Input resistance (MΩ)** | 319 ± 7 | 361 ± 39 | **608 ± 64** | **569 ± 26** | 350 ± 36 | 387 ± 22 | **201 ± 13** |
|  | **FS-*Pvalb*** <<< RS, IB, Burst. **Vip**, Adapt. *Vip*, Adapt. *Sst*, Adapt. *Npy*  RS, Adapt. *Sst*, Adapt. *Npy* <<< **Adapt. *Vip***; IB << **Adapt. *Vip***  RS <<< **Burst. *Vip*;** Adapt. *Sst*, Adapt. *Npy* << **Burst. *Vip*** | | | | | | |
| **(3) Time constant (ms)** | 32.4 ± 1.4 | 33.5 ± 3.1 | 31.0 ± 2.6 | 27.8 ± 1.5 | 28.3 ± 3.3 | 28.1 ± 1.5 | **16.2 ± 1.0** |
|  | **FS-*Pvalb*** <<< RS, IB, Burst. *Vip*, Adpat. *Vip*, Adapt. *Npy*; **FS-*Pvalb*** << Adapt. *Sst* | | | | | | |
| **(4) Membrane capacitance (pF)** | **110.7 ± 4.1** | 95.6 ± 5.9 | **56.3 ± 4.0** | **51.1 ± 2.8** | 82.8 ± 4.7 | 76.8 ± 3.3 | 83.8 ± 3.6 |
|  | **Adapt. *Vip*** <<< RS, IB, Adapt. *Sst*, Adapt. *Npy*, FS-*Pvalb*  **Burst. *Vip*** <<< RS, IB, Adapt. *Sst*, FS-*Pvalb*; **Burst. *Vip*** << Adapt. *Npy*  Adapt. *Sst*, Adapt. *Npy*, FS-*Pvalb* <<< **RS** | | | | | | |
| **(5) Sag index (%)** | 14.1 ± 1.1 | **26.1 ± 2.3** | 5.7 ± 0.9 | 8.3 ± 0.7 | **24.9 ± 2.5** | 9.5 ± 0.6 | 9.0 ± 0.9 |
|  | Burst. *Vip*, Adapt. *Vip*, Adapt. *Npy*, FS-*Pvalb* <<< **IB, Adapt. *Sst***; RS << **IB**, **Adapt. *Sst***  Burst. *Vip* <<< RS; Burst. *Vip* << Adapt. *Npy*  Adapt. *Vip* << RS; FS-*Pvalb* < RS | | | | | | |

n, number of cells, < significantly smaller with P ≤ 0.05; << significantly smaller with P ≤ 0.01; <<< significantly smaller with P ≤ 0.001
