# Supplementary material for: Lactate is an energy substrate for rodent cortical neurons and enhances their firing activity
Source: eLife. 2021 Nov 12;10:e71424. doi: 10.7554/eLife.71424 (PMC8651295; doi:10.7554/eLife.71424)
Supplement: Supplementary file 4. [file elife-71424-supp4.docx]

**Supplementary file 4. Just above threshold properties of different neuronal types**

|  | **RS** | **IB** | **Burst. *Vip*** | **Adapt. *Vip*** | **Adapt. *Sst*** | **Adapt. *Npy*** | **FS-*Pvalb*** |
| --- | --- | --- | --- | --- | --- | --- | --- |
|  | **n = 63** | **n = 10** | **n = 27** | **n = 59** | **n = 24** | **n = 56** | **n = 38** |
| **(6) Rheobase (pA)** | 52.0 ± 4.2 | 39.2 ± 7.5 | 25.8 ± 4.1 | **14.5 ± 2.3** | **14.0 ± 6.2** | 40.8 ± 4.1 | **99.3 ± 8.4** |
|  | RS, IB, Burst. *Vip*, Adapt *Vip*, Adapt. *Sst*, Adapt. *Npy* <<< **FS-*Pvalb***  **Adapt. *Vip*** <<< RS, Adapt. *Npy*; **Adapt. *Vip*** << IB  **Adapt. *Sst*** <<< RS; **Adapt. *Sst*** << Adapt. *Npy*  Burst. *Vip* << RS | | | | | | |
| **(7) First spike latency (ms)** | 151.3 ± 9.5 | 147.9 ± 25.2 | 102.2 ± 19.3 | 109.4 ± 10.7 | 144.3 ± 27.2 | 224.9 ± 26.1 | **348.3 ± 42.2** |
|  | Burst. *Vip*, Adapt. *Vip* <<< **FS-*Pvalb***; RS, Adapt. *Sst* << **FS-*Pvalb***  Adapt. *Vip* <<< RS, Burst. *Vip* << RS  Adapt. *Vip*, Burst. *Vip* << Adapt. *Npy* | | | | | | |
| **(8) Adaptation (Hz/s)** | -9.8 ± 2.9 | **-168.8 ± 26.0** | **-658.4 ± 412.9** | -8.3 ± 3.0 | -14.4 ± 4.1 | **-1.9 ± 0.7** | **20.9 ± 22.2** |
|  | **IB, Burst. *Vip*** <<< RS, Adapt. *Vip*, Adapt. *Sst*, Adapt. *Npy*, FS-*Pvalb*  RS, Adapt. Vip, Adapt. Sst << **Adapt. Npy, FS-Pvalb** | | | | | | |
| **(9) Minimal steady state frequency (Hz)** | 8.2 ± 1.3 | **66.2 ± 4.8** | **61.4 ± 8.4** | 11.6 ± 0.9 | 13.3 ± 2.3 | 6.8 ± 0.4 | 15.1 ± 1.1 |
|  | RS, Adapt. *Vip*, Adapt. *Sst*, Adapt. *Npy*, FS-*Pvalb* <<< **IB, Burst. *Vip***  **Adapt. *Npy*** <<< Adapt. *Vip*, FS-*Pvalb*, **Adapt. *Npy*** << Adapt. *Sst*  RS <<< Adapt. *Vip* << FS-*Pvalb* | | | | | | |

n, number of cells; < significantly smaller with P ≤ 0.05; << significantly smaller with P ≤ 0.01; <<< significantly smaller with P ≤ 0.001
