# Supplementary material for: Lactate is an energy substrate for rodent cortical neurons and enhances their firing activity
Source: eLife. 2021 Nov 12;10:e71424. doi: 10.7554/eLife.71424 (PMC8651295; doi:10.7554/eLife.71424)
Supplement: Supplementary file 5. [file elife-71424-supp5.docx]

**Supplementary file 5. Firing properties of different neuronal types**

|  | **RS** | **IB** | **Burst. *Vip*** | **Adapt. *Vip*** | **Adapt. *Sst*** | **Adapt. *Npy*** | **FS-*Pvalb*** |
| --- | --- | --- | --- | --- | --- | --- | --- |
|  | **n = 63** | **n = 10** | **n = 27** | **n = 59** | **n = 24** | **n = 56** | **n = 38** |
| **(10) Amplitude accommodation (mV)** | **18.5 ± 1.1** | **36.7 ± 3.1** | 9.9 ± 1.4 | 5.7 ± 0.5 | 3.8 ± 0.6 | 8.7 ± 0.9 | **2.0 ± 0.3** |
|  | Burst. *Vip*, Adapt. *Vip*, Adapt. *Sst*, Adapt. *Npy*, FS-*Pvalb* <<< **RS** <<< **IB**  **FS-*Pvalb*** <<< Burst. *Vip*, Adapt. *Vip*, Adapt *Npy*; FS-*Pvalb* < Adapt. *Sst*  Adapt. *Sst* << Burst. *Vip*, Adapt. *Npy*; Adapt. *Sst* < Adapt. *Vip*  Adapt. *Vip* < Burst. *Vip*, Adapt. *Npy* | | | | | | |
| **(11) Amplitude of early adaptation (Hz)** | 85.2 ± 3.9 | 96.1 ± 6.4 | 79.9 ± 8.3 | 76.1 ± 4.3 | 76.9 ± 7.4 | 84.6 ± 3.3 | **47.9 ± 3.1** |
|  | **FS-*Pvalb*** <<< RS, IB, Burst. *Vip*, Adapt. *Vip*, Adapt. *Npy*; **FS-*Pvalb*** << Adapt. *Sst* | | | | | | |
| **(12) Time constant of early adaptation (ms)** | **19.6 ± 0.8** | **38.5 ± 5.2** | 25.8 ± 2.4 | 26.0 ± 1.3 | **38.6 ± 3.7** | 25.6 ± 1.0 | 21.1 ± 3.1 |
|  | **RS** <<< IB, Adapt. *Sst*, Adapt *Npy*; **RS** << Adapt. *Vip*, **RS** < Burst. *Vip*  Adapt. *Npy*, FS-*Pvalb* <<< **Adapt. *Sst***; Adapt. *Vip* << **Adapt. *Sst***; Burst. *Vip* < **Adapt. *Sst***  Adapt. Npy << **IB**; Burst. Vip, Adapt. Vip, FS-Pvalb < **IB**  FS-*Pvalb* < Adapt. *Vip*, Adapt. *Npy* | | | | | | |
| **(13) Late adaptation (Hz/s)** | -16.2 ± 1.4 | **-3.6 ± 1.8** | -204.1 ± 181.7 | -35.7 ± 2.9 | -24.8 ± 2.9 | -19.5 ± 1.2 | -29.4 ± 2.1 |
|  | **IB** << RS; **IB** <<< Burst. *Vip*, Adapt. *Vip*, Adapt. *Sst*, Adapt . *Npy*, FS-*Pvalb*  RS, Adapt. *Npy* <<< Adapt. *Vip*, FS-*Pvalb* | | | | | | |
| **(14) Maximal steady state frequency (Hz)** | **37.2 ± 1.7** | **24.4 ± 2.5** | 68.7 ± 7.3 | 80.0 ± 5.6 | 72.8 ± 5.2 | 56.4 ± 2.0 | **139.9 ± 6.8** |
|  | **IB** << **RS** <<< Burst. *Vip*, Adapt. *Vip*, Adapt. *Sst*, Adapt. *Npy* <<< **FS-*Pvalb***  Adapt. *Npy* << Adapt. *Vip*; Adapt. *Npy* < Adapt. *Sst* | | | | | | |

n, number of cells; < significantly smaller with P ≤ 0.05; << significantly smaller with P ≤ 0.01; <<< significantly smaller with P ≤ 0.001
