# Supplementary material for: Lactate is an energy substrate for rodent cortical neurons and enhances their firing activity
Source: eLife. 2021 Nov 12;10:e71424. doi: 10.7554/eLife.71424 (PMC8651295; doi:10.7554/eLife.71424)
Supplement: Supplementary file 6. [file elife-71424-supp6.docx]

**Supplementary file 6. Action potentials properties of different neuronal types**

|  | **RS** | **IB** | **Burst. *Vip*** | **Adapt. *Vip*** | **Adapt. *Sst*** | **Adapt. *Npy*** | **FS-*Pvalb*** |
| --- | --- | --- | --- | --- | --- | --- | --- |
|  | **n = 63** | **n = 10** | **n = 27** | **n = 59** | **n = 24** | **n = 56** | **n = 38** |
| **(15) First spike amplitude (mV)** | 92.1 ± 1.2 | 90.8 ± 1.5 | 94.3 ± 2.3 | 90.9 ± 1.3 | 94.9 ± 2.5 | 89.0 ± 1.3 | **80.9 ± 1.4** |
|  | **FS-*Pvalb*** <<< RS, Burst. *Vip*, Adapt. *Vip*, Adapt. *Sst*, Adapt. *Npy*; **FS-*Pvalb*** << IB | | | | | | |
| **(16) First spike duration (ms)** | **1.4 ± 0.0** | 1.4 ± 0.1 | 0.9 ± 0.1 | 0.9 ± 0.0 | 0.9 ± 0.1 | 1.1 ± 0.0 | **0.6 ± 0.0** |
|  | **FS-*Pvalb*** <<< Burst. *Vip*, Adapt. *Vip*, Adapt. *Sst*, Adapt. *Npy* <<< **RS**  **FS-*Pvalb*** <<< **IB**; Burst. *Vip*, Adapt. *Vip*, Adapt. *Sst* <<< **IB**, Adapt. *Npy* << **IB**  Burst. *Vip*, Adapt. *Vip* <<< Adapt. *Npy*; Adapt. *Sst* < Adapt. *Npy* | | | | | | |
| **(17) Second spike amplitude (mV)** | 89.6 ± 1.3 | **68.4 ± 1.8** | **75.5 ± 2.1** | 87.1 ± 1.4 | 91.9 ± 2.2 | 86.0 ± 1.3 | **79.9 ± 1.5** |
|  | **IB, Burst. *Vip*** <<< RS, Adapt. *Vip*, Adapt. *Sst*, Adapt. *Npy*, **IB** << **FS-*Pvalb***  **FS-*Pvalb*** <<< RS, Adapt. *Sst*; **FS-*Pvalb*** << Adapt. *Vip*, Adapt. *Vip* | | | | | | |
| **(18) Second spike duration (ms)** | **1.5 ± 0.0** | **1.8 ± 0.1** | 1.0 ± 0.1 | 0.9 ± 0.0 | 1.0 ± 0.1 | 1.2 ± 0.0 | **0.6 ± 0.0** |
|  | **FS-*Pvalb*** <<< Burst. *Vip*, Adapt. *Vip*, Adapt. *Sst*, Adapt. *Npy* <<< **RS** << **IB**  Adapt. *Vip* <<< Adapt. *Npy*; Burst. *Vip*, Adapt. *Sst* < Adapt. *Npy* | | | | | | |
| **(19) Amplitude Reduction (%)** | 2.8 ± 0.4 | **24.7 ± 1.5** | **19.7 ± 1.7** | 4.2 ± 0.4 | 2.9 ± 0.7 | 3.3 ± 0.6 | **1.2 ± 0.9** |
|  | RS, Adapt. *Vip*, Adapt. *Sst*, Adapt. *Npy*<<< **IB, Burst. *Vip***  **FS-*Pvalb*** <<< Adapt.*Vip*; **FS-*Pvalb*** < RS, Adapt. *Npy*, RS < Adapt. *Vip* | | | | | | |
| **(20) Duration Increase (%)** | 4.8 ± 0.4 | **31.3 ± 3.4** | **14.5 ± 1.8** | 4.5 ± 0.5 | 4.9 ± 0.6 | 7.6 ± 0.9 | **0.8 ± 0.5** |
|  | **FS-*Pvalb*** <<< Adapt. *Vip*, Adapt. *Sst*, Adapt. *Npy*<<< **Burst .*Vip*** <<< **IB**  Adapt. *Vip* << Adapt. *Npy*, RS < Adapt. *Npy* | | | | | | |

n, number of cells; < significantly smaller with P ≤ 0.05; << significantly smaller with P ≤ 0.01; <<< significantly smaller with P ≤ 0.001
