# Supplementary material for: Lactate is an energy substrate for rodent cortical neurons and enhances their firing activity
Source: eLife. 2021 Nov 12;10:e71424. doi: 10.7554/eLife.71424 (PMC8651295; doi:10.7554/eLife.71424)
Supplement: Supplementary file 7. [file elife-71424-supp7.docx]

**Supplementary file 7. AH and AD properties of different neuronal types**

|  | **RS** | **IB** | **Burst. *Vip*** | **Adapt. *Vip*** | **Adapt. *Sst*** | **Adapt. *Npy*** | **FS-*Pvalb*** |
| --- | --- | --- | --- | --- | --- | --- | --- |
|  | **n = 63** | **n = 10** | **n = 27** | **n = 59** | **n = 24** | **n = 56** | **n = 38** |
| **(21) First spike, fast AH (mV)** | -7.2 ± 0.3 | -7.0 ± 0.8 | -12.3 ± 0.7 | -14.7 ± 0.6 | -14.2 ± 1.1 | -14.5 ± 0.7 | **-23.7 ± 0.6** |
|  | **FS-*Pvalb*** <<< Burst. *Vip*, Adapt. *Vip*, Adapt. *Sst*, Adapt. *Npy* <<< **RS, IB** | | | | | | |
| **(22) first spike AD (mV)** | 1.0 ± 0.2 | 0.0 ± 0.0 | 0.1 ± 0.1 | **5.2 ± 0.5** | **3.1 ± 0.6** | 0.3 ± 0.1 | 0.8 ± 0.3 |
|  | RS, IB, Burst. *Vip*, Adapt. *Npy*, FS-*Pvalb* <<< **Adapt. *Vip***  Burst. *Vip*, Adapt. *Npy* <<< **Adapt. *Sst*,** RS, IB, FS-*Pvalb* <<< **Adapt. *Sst*** | | | | | | |
| **(23) First spike, medium AP (mV)** | **-14.5 ± 0.4** | **0.0 ± 0.0** | **-0.3 ± 0.3** | -10.9 ± 0.6 | -8.2 ± 1.4 | -11.6 ± 1.0 | -4.5 ± 1.4 |
|  | **RS** <<< IB, Burst. *Vip*, Adapt. *Vip*, Adapt. *Sst*, FS-*Pvalb*  Adapt. *Vip*, Adapt. *Npy* <<< **IB, Burst. *Vip***, Adapt. *Sst* <<< **Burst. *Vip***, Adapt. *Sst* << **IB**  Adapt .*Vip* <<< FS-*Pvalb*, Adapt. *Npy* << FS-*Pvalb* | | | | | | |
| **(24) First spike, fast AP latency (ms)** | **6.5 ± 0.3** | 4.4 ± 0.2 | 2.8 ± 0.2 | 3.2 ± 0.2 | 3.3 ± 0.2 | 5.2 ± 0.2 | 2.7 ± 0.2 |
|  | Burst. *Vip*, Adapt. *Vip*, Adapt. *Sst*, FS-*Pvalb* <<< Adapt. *Npy* << **RS**  Burst. *Vip*, FS-*Pvalb* <<< IB; Adapt. *Vip* << IB, IB < Adapt. *Sst*, **RS** | | | | | | |
| **(25) First spike, AD latency (ms)** | 4.6 ± 0.7 | 0.0 ± 0.0 | 0.4 ± 0.4 | **9.9 ± 0.7** | **7.5 ± 1.3** | 3.1 ± 0.6 | 1.2 ± 0.4 |
|  | RS, IB, Burst. *Vip*, Adapt. *Npy*, FS-*Pvalb* <<< **Adapt. *Vip***  Burst. *Vip*, FS-*Pvalb* <<< **Adapt. *Sst***; IB, Adapt. *Npy* < **Adapt. *Sst*** | | | | | | |
| **(26) First spike, medium AH latency (ms)** | **63.1 ± 3.0** | 0.0 ± 0.0 | 0.5 ± 0.5 | **30.0 ± 2.5** | 23.1 ± 3.9 | 18.5 ± 1.9 | 2.2 ± 0.7 |
|  | IB, Burst. *Vip*, Adapt. *Vip*, Adapt. *Sst*, Adapt. *Npy*, FS-*Pvalb* <<< **RS**  IB, Burst. *Vip*, FS-*Pvalb* <<< Adapt. *Npy* << **Adapt. *Vip***  Burst. *Vip*, Adapt. *Npy* <<< Adapt. *Sst*, IB << Adapt. *Sst* | | | | | | |
| **(27) Second spike, fast AH (mV)** | **-7.7 ± 0.3** | **-8.7 ± 0.8** | -14.3 ± 0.7 | -15.9 ± 0.6 | -13.8 ± 1.1 | -15.3 ± 0.8 | **-24.0 ± 0.6** |
|  | **FS-*Pvalb*** <<< Burst. *Vip*, Adapt. *Vip* Adapt. *Sst*, Adapt. *Npy* <<< **RS**  **FS-*Pvalb***, Burst. *Vip*, Adapt. *Vip* Adapt. *Npy* <<< **IB**, Adapt. *Sst*< **IB** | | | | | | |
| **(28) Second spike AD (mV)** | 0.2 ± 0.0 | 0.4 ± 0.2 | 2.8 ± 0.7 | **4.3 ± 0.4** | 2.8 ± 0.5 | 0.1 ± 0.0 | 0.8 ± 0.3 |
|  | RS, IB, Adapt. *Npy*, FS-*Pvalb* <<< **Adapt. *Vip***  RS, Adapt *Npy* <<< Adapt. *Sst*; FS-*Pvalb* << Adapt. *Sst* | | | | | | |
| **(29) Second spike, medium AH (mV)** | **-17.3 ± 0.5** | **-24.9 ± 0.9** | -6.9 ± 1.4 | -12.7 ± 0.6 | -8.1 ± 1.3 | -13.2 ± 1.1 | -4.0 ± 1.3 |
|  | **IB** <<< **RS** <<< Burst. *Vip*, Adapt. *Vip* Adapt. *Sst*, Adapt. *Npy*, FS-*Pvalb*  Adapt. *Npy*, Adapt *Vip* << FS-*Pvalb*; Adapt. *Npy* << Burst. *Vip*; Adapt *Vip* < Burst. *Vip*  Adapt. *Npy*, Adapt *Vip* < Adapt. *Sst*; Adapt. *Sst* < FS-*Pvalb* | | | | | | |
| **(30) Second spike, fast AH latency (ms)** | **6.9 ± 0.3** | **7.5 ± 0.5** | 3.5 ± 0.3 | 3.6 ± 0.2 | 3.5 ± 0.3 | 5.6 ± 0.2 | **2.8 ± 0.2** |
|  | **FS-*Pvalb*** < Burst. *Vip*, Adapt. *Vip*, Adapt. *Sst* <<< Adapt. *Npy* << **RS, IB** | | | | | | |
| **(31) Second spike AD latency (ms)** | 1.8 ± 0.4 | 6.6 ± 1.6 | 4.3 ± 1.0 | **8.7 ± 0.6** | **7.5 ± 1.2** | 1.6 ± 0.4 | 0.9 ± 0.3 |
|  | RS, Adapt. *Npy*, FS-*Pvalb*<<< **Adapt. *Vip*, Adapt. *Sst***; Burst. *Vip* < **Adapt. *Vip***  RS, Adapt. *Npy*, FS-*Pvalb*< IB | | | | | | |
| **(32) Second spike, medium AH latency (ms)** | **62.9 ± 2.9** | **100.0 ± 8.0** | 21.9 ± 4.8 | 28.8 ± 2.1 | 24.0 ± 3.8 | 19.4 ± 2.2 | **2.1 ± 0.7** |
|  | **FS-*Pvalb*** <<< Adapt.*Vip*, Adapt. *Sst*, Adapt. *Npy* <<< **RS** <<< **IB**  Burst. *Vip* <<< **RS, IB;** FS-*Pvalb* < Burst. *Vip*; Adapt. *Npy* << Adapt. *Vip* | | | | | | |

n, number of cells; < significantly smaller with P ≤ 0.05; << significantly smaller with P ≤ 0.01; <<< significantly smaller with P ≤ 0.001
